# Multiple analyses of various factors affecting the plantlet regeneration of *Picea mongolica* (H. Q. Wu) W.D. Xu from somatic embryos

**DOI:** 10.1038/s41598-021-83948-w

**Published:** 2021-03-23

**Authors:** Jia Yan, Peng Peng, Guozhen Duan, Tao Lin, Yu´e Bai

**Affiliations:** 1 Institute of Forest Tree Genetic Breeding, Forestry College, Inner Mongolia Agricultural University, Xinjian Street 275, Hohhot City, Inner Mongolia China; 2 Institute of Forestry, Academy of Agricultural and Forestry Sciences, Qinghai University, Ning da Road 251, Xining City, Qinghai Province China

**Keywords:** Molecular biology, Plant sciences

## Abstract

*Picea mongolica*, a native species with excellent industrial wood quality and strong sand-fixing capacity, may be utilized in construction of urban green spaces in arid areas in China. However, now the sustainability of the ecosystems where this species grows is at serious risk due to a lack of natural regeneration. In this study, we developed an efficient regeneration system and comprehensively analyzed various factors affecting somatic embryogenesis (SE) using zygotic embryos as explants. We identified the optimal plant growth regulators (PGRs) performance and the best donor trees (k81) for the generation of somatic embryos (SEMs). Additionally, we confirmed that the positive developmental window of SEMs initiation was at the end of July to early August, which is when zygotic embryos was at the late embryogeny. In this time period, specific transcripts associated with the regulation of epigenetic modifications, plant hormone-related genes, and embryonic development-related transcription factors play important roles for early SEMs initiation. These results may provide a valuable resource for vegetative propagation of *Picea mongolica*. Our results may help to establish a reliable protocol for plantlet regeneration, which may facilitate urban greening applications and industrialization in arid areas.

## Introduction

Somatic embryogenesis (SE) is a powerful in vitro technique that has been demonstrated significant benefits in clonal mass planta propagation. It is a process of differentiation in which cells with asymmetric division ability resembling a zygotic embryo develops into planta through a series of structural, physiological, and molecular events. In general, the process of SE consists of ordered steps: initiation, proliferation, maturation and plant regeneration^1,2^. The pathways of SE could be divided into direct and indirect types based on the occurrence mode with or without an intervening callus phase from induction to maturation^3^. Since the publication of the original report describing SE in *Daucus carota*, this technique has been employed in various plant species, including herbs, shrubs, lianas and trees^4–8^. The embryogenic cultures (EC) pathway is propagated in many various plant species^9,10^. In 1985, SEMs was produced for the first time in a coniferous species, *Picea abies*^11^. In subsequent years, SE technology has been successfully applied in many coniferous species^12–14^. Numerous studies have successfully regenerated SEMs by selecting the appropriate explant type^15^, species/genotype^16^ and plant growth regulator (PGR) in coniferous plants SE studies^17,18^. Despite the advances made in many coniferous species, the SE protocol has not been reported in *Picea mongolica.*

Embryogenic cultures can be generated from a variety of tissues, including zygotic embryos and shoots in many coniferous species^19^. However, a major drawback of the current SE methodology has been reported that the induction of SEMs is difficult with mature zygotic embryos. In fact, ensuring that the embryo developmental stage of zygotic embryo explants is critical to the outcome of propagation in vitro, and the time window for the positive response is often short^20^. Additionally, PGRs are also critical in the initiation, maintenance, and maturation of SEMs^21–23^. The findings of previous studies indicate that PGRs, especially auxin and cytokinin, are associated with the initiation and proliferation of embryogenic tissue. Abscisic acid (ABA) and PEG, osmotic pressure-regulating agents, also serve a significant function in the maturation of SEMs. These agenysy are critical for ensuring the rapid accumulation of early SEMs, and generation high yield, high-quality cotyledonary SEMs^24^. Previous studies indicate that the transition from the pro-embryogenic mass to the embryo during SE in Norway spruce is initiated by withdrawal of plant growth regulators^25^.

*Picea* (spruce) is the third largest genus after *Pinus* and *Abies* in the *Pinaceae* family^26^. *P. mongolica* (H. Q. Wu) W. D. Xu. is a rare spruce that covers only approximately 1947 hectares and forms a spectacular and unique forest landscape on the sandy grassland distributed on the eastern edge of the Hun Shandake Sandy Land of Inner Mongolia in China. Due to the strong sand-fixing capacity of this species, *P. mongolica* is a key windbreak vegetation for preventing the desertification of land in northern China. In recent years, their ecosystems where this plant grows have been at serious risk due to the lack of natural regeneration. The reproduction of *P. mongolica* is limited by insufficient seeds, plant age, the location of the mother tree and the poor quality of cuttings due to fire and pests^27^. Therefore, the SEMs reproduction may be an effective pathway to enhance *P. mongolica* yield. This method not only ensures mass propagation of high-quality trees but also ensures that the trees receive its high-value genotypes from their parents^28^.

To improve the efficiency of SE, we comprehensively analyzed the function of several factors, including PGRs, zygotic embryo collection times and maternal tree genotypes in SE regeneration pathway of *P. mongolica*. Utilizing analyses and transcriptomic data, we improved the efficiency of the ECs initiation, proliferation, and SEMs maturation. Our work provides an effective regeneration technology, important molecular basis and gene resources for elucidating the somatic embryogenesis mechanism in *P. mongolica*. This study helps to establish a foundation for future research and presents a promising technology for the propagation of *P. mongolica* at Hun Shandake Sandy Land nature reserve in China.

## Results

### SEMs regeneration from zygotic embryos of *Picea mongolica*

**Figure 1:**
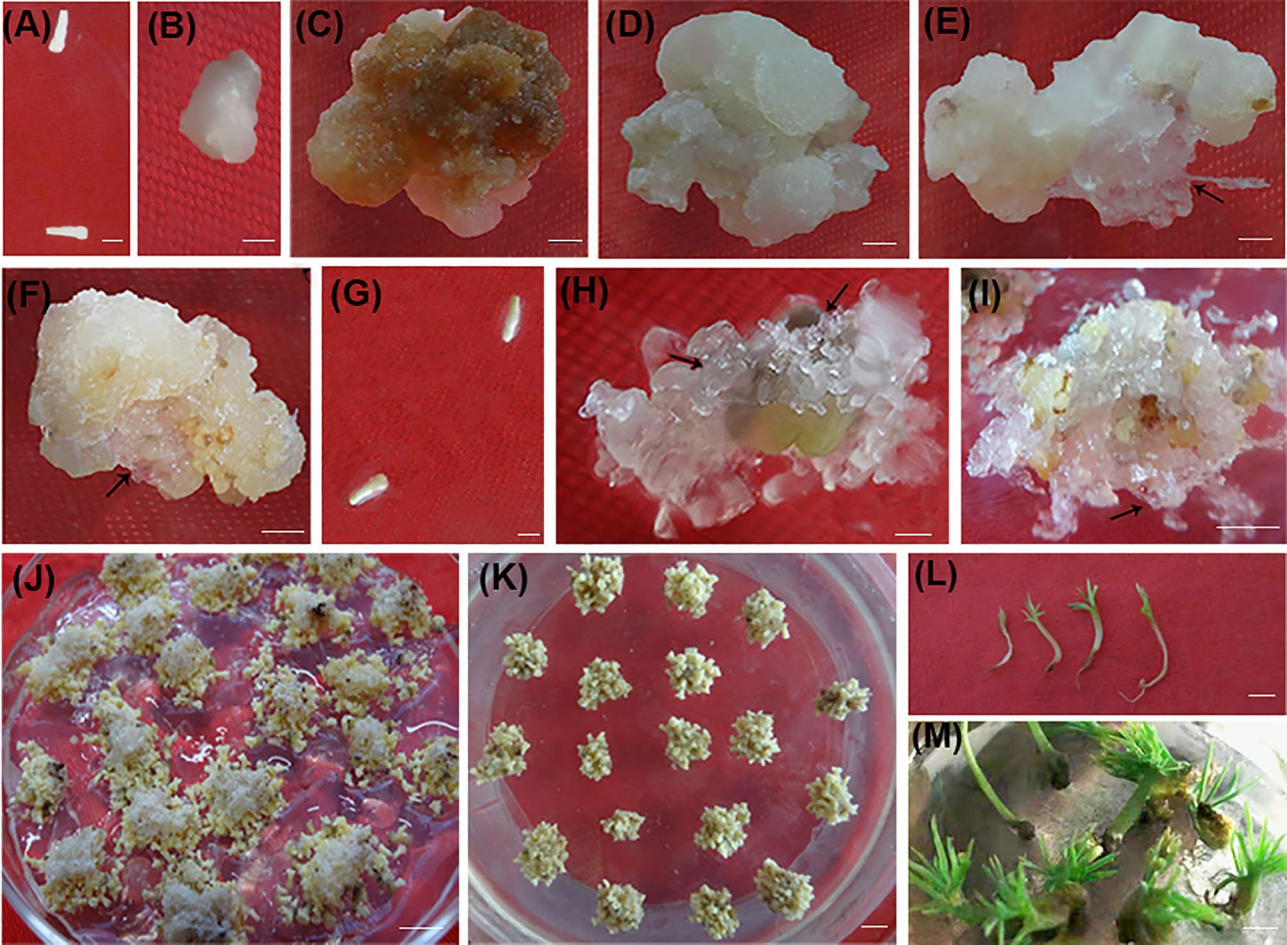
Somatic embryogenesis from zygotic embryos. (**A**–**F**) Induction of embryogenic tissues on zygotic embryos from mature seeds. Bars: 0.5 cm. (**A**) Cotyledonary zygotic embryos from mature seeds, (**B**–**E**) induction of calli on zygotic embryos from mature seed after 1 week (**B**), 4 weeks (**C**–**E**). (**C**) Non-embryogenic callus of browning with cells accumulating phenolic compounds. (**D**) Non-embryogenic callus without phenolics, (**E**) Embryogenic culture initiated on the calli mass. The arrow points to the filamentous and transparent embryogenic cultures. (**F**) proliferation of embryogenic culture with some pieces of non-embryogenic calli. (**G**–**I**) Induction of embryogenic tissues on zygotic embryos from immature seeds. Bars: 0.5 cm. (**G**) zygotic embryos from immature seeds. (**H**) Embryogenic culture initiated on immature zygotic embryos. (**I**) Proliferation of embryogenic cultures. (**J**) Differentiation of embryogenic tissues with maturation of somatic embryos on the proliferating ECs. (**K**) Maturation of somatic embryos on the maturation medium. (**L**) Germination. Bars: 0.5 cm. (**M**) Seedlings produced by germination from somatic embryos. Bars: 1 cm.

To obtain SEMs of *P. mongolica*, zygotic embryos from mature and immature seeds were collected separately and placed on different induction media to generate ECs. When mature embryos were used as explant, the surviving embryos gradually dedifferentiated into embryogenic and non-embryogenic tissues (Fig. 1A–E). After 1-week, a small white or pale yellow, shiny, and soft nodular structure appeared on the surface of zygotic embryos (Fig. 1B). After 3 weeks, some EC tissues were organized into transparent and filamentous small clumps on calli mass (Fig. 1E). Non-embryogenic cultures began to turn dark brown with cells that accumulated phenolic compounds or white tissues without phenolics (Fig. 1C,D). The browned tissues soon became necrotic. Subsequently, the ECs with some pieces of non-embryogenic calli could continue to proliferate (Fig. 1F), but many of these tissues have similar browning phenomenon at the later growth. Moreover, the EC induction rate was very low. To improve the EC induction efficiency, we obtained immature embryos as explants (Fig. 1G–I). After 3 weeks, transparent and filamentous ECs appeared and multiply proliferated on calli mass (Fig. 1H,I). Embryogenic cultures were transferred and differentiated culture; some SEMs appeared around clumps of still proliferating EC after 3 weeks (Fig. 1J). Finally, mature cotyledonary embryos were obtained on the maturation medium after 3 weeks (Fig. 1K). After 2 weeks of drying, SEMs could germinate into seedlings with roots emergence after a week of dark cultivation; cotyledons grow on the top of the epicotyl after 2 weeks of light cultivation (Fig. 1L). Subsequently, through 2 weeks growth in incubator culture (Fig. 1M), they were transplanted into the substrate with soil for further cultivation.

**Figure 2:**
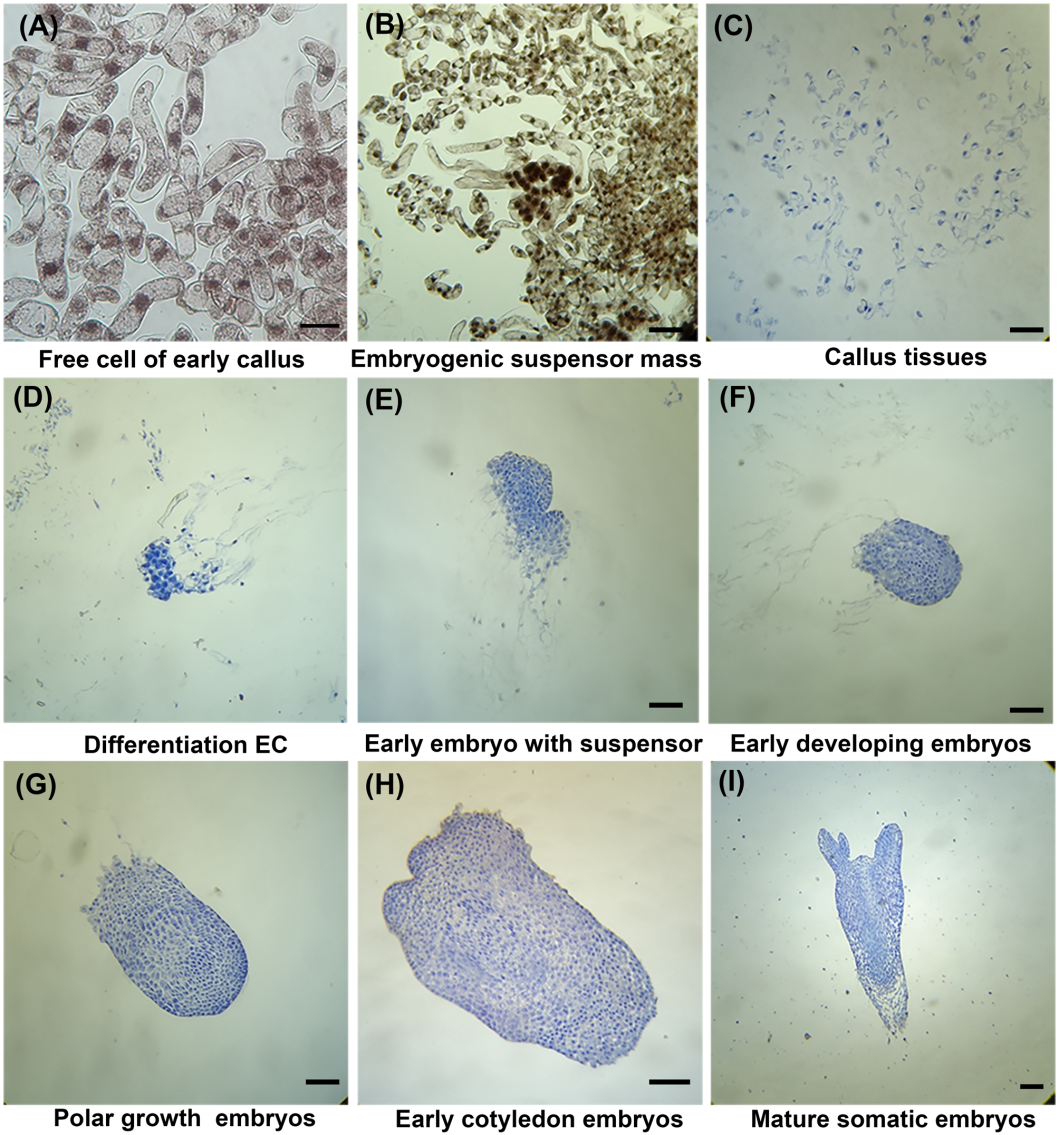
Development stages of somatic embryos in *Picea mongolica.* (**A**, **B**) Early embryogenic cultures stained with gentian violet. (**A**) Free elongated suspensor cells from very early initiation of callus after 1 weeks EC induction; Bar: 100 μm. (**B**) Early embryogenic suspensor mass with some free suspensor cells after 2 weeks EC induction; Bar: 50 μm. (**C**–**I**) Development stages of somatic embryos with cell staining after paraffin sectioning. (**C**) Free suspensor cells in early induced the embryogenic cultures after 2 weeks; Bar: 50 μm. (**D**) Early differentiated embryogenic cultures mass after 3 weeks; Bar: 0.5 mm. (**E**) Embryogenic suspensor mass; Bar: 0.5 mm. (**F**) Early developing embryo disappeared suspensor; Bar: 0.5 mm. (**G**) Developing embryo with polar growth; Bar: 0.5 mm. (**H**) Early cotyledon embryos; (**I**) Mature cotyledon embryos. Bar: 0.5 mm.

To directly detect whether SEMs was initiated, samples of induced tissues were stained with 1% gentian violet staining buffer. As shown in Fig. 2A, there were only just free elongated suspensor cells from very early initiation of calli after 1 weeks EC induction. With increasing of induction time, embryogenic suspensor mass (ESM) was formed around with free suspensor cells 1 week later (Fig. 2B). Additionally, paraffin sections result also showed the same irregular free suspensor cells with the embryogenic and no-embryogenic cultures in early EC induction (Fig. 2C). With the proliferation and differentiation of ECs, we observed the whole development process of SEMs in vitro through paraffin sections assay (Fig. 2D–I). After 1 week of ECs proliferation, the early SEMs gradually developed to form hemispherical meristems (Fig. 2D). After 2 weeks, the embryos had already organized into developing embryos and the suspensor cells gradually became apoptotic (Fig. 2E,F). Subsequently, the embryo suspensor cells almost died and completely disappeared in the following week; at this point, the embryos were already quite well-organized developing embryos (Fig. 2F). Later, generated SEMs began to polar grow with denser apical cells and loose basal cells. The meristem cells gradually grew globularly and developed into late embryogeny stage after 4 weeks (Fig. 2G,H). After approximately 5 weeks, the tops of the embryos gradually protruded and developed into early cotyledons embryos (Fig. 2H). Mature SEMs were regularly arranged, and cotyledons were grown in an orderly manner (Fig. 2I).

### Plant growth regulators play important roles in EC initiation

To obtain the most valuable system for ECs induction, two efficient somatic embryogenesis systems were developed in *P. mongolica.* Mature cotyledons embryos were obtained and grown on MEC1-MEC9 media with different concentrations of PGRs (auxin and cytokinin) (Table 1). Our statistical results showed that the percentage of callus induction was increased to 72% in MEC1 (Table 1), but it is readily turned brown in the following cultivation. Next, we added cytokinin Kinetin (KT) to the LM medium with 6-benzylaminopurine hydrochloride (6-BA) and 2,4-Dichlorophenoxyacetic acid (2,4-D) (MEC2). After 4 weeks, the statistical results showed that the induction percentage was up to 78%, but the generated calli were non-embryogenic tissues; it was mostly milky white, tightly blocked tissues and readily turn browned. Moreover, the ECs were still not generated by adding NAA or decreasing 2,4-D to reduce callus browning in mediums MEC3-6. (Table 1). After that, we decreased the concentration of KT (MEC9) when the inducted callus was transferred to MEC medium and to further cultivate. The transparent and filamentous embryogenic cultures were generated at the edge or surface of the callus after 4 weeks, but the induction rate was only 12% (Table 1).

**Table 1:** The medium combinations for inducing EC from mature zygotic embryo.

Code	Initial culture			Transfer culture	
	Basal medium + PGRs (mg L^−1^)	Frequency of callus (%)	Frequency of EC (%)	Basal medium + PGRs (mg L^−1^)	Frequency of EC (%)
MEC1	LM + 2.0 (6-BA) + 4.0 (2,4-D)	72	0	LM + 2.0 (6-BA) + 4.0 (2,4-D)	0
MEC2	LM + 2.0 (6-BA) + 1.0 (KT) + 4.0 (2,4-D)	78	0	LM + 2.0 (6-BA) + 1.0 (KT) + 4.0 (2,4-D)	0
MEC3	LM + 2.0 (6-BA) + 1.0 (KT) + 4.0 (2,4-D) + 1.0 (NAA)	66	0	LM + 2.0 (6-BA) + 1.0 (KT) + 4.0 (2,4-D) + 1.0 (NAA)	0
MEC4	LM + 2.0 (6-BA) + 1.0 (KT) + 4.0 (2,4-D) + 2.0 (NAA)	74	0	LM + 2.0 (6-BA) + 1.0 (KT) + 4.0 (2,4-D) + 2.0 (NAA)	0
MEC5	LM + 2.0 (6-BA) + 1.0 (KT) + 4.0 (2,4-D) + 3.0 (NAA)	62	0	LM + 2.0 (6-BA) + 1.0 (KT) + 4.0 (2,4-D) + 3.0 (NAA)	0
MEC6	LM + 2.0 (6-BA) + 1.0 (KT) + 3.0 (2,4-D) + 3.0 (NAA)	68	0	LM + 3.0 (6-BA) + 1.0 (KT) + 3.0 (2,4-D) + 3.0 (NAA)	0
MEC7	LM + 2.0 (6-BA) + 1.0 (KT) + 4.0 (2,4-D)	78	0	1/2 LM + 2.0 (6-BA) + 4.0 (2,4-D)	0
MEC8	LM + 2.0 (6-BA) + 1.0 (KT) + 4.0 (2,4-D)	78	0	1/2 LM + 2.0 (6-BA) + 3.0 (NAA)	0
MEC9	LM + 2.0 (6-BA) + 1.0 (KT) + 4.0 (2,4-D)	78	0	1/2 LM + 1.0 (6-BA) + 0.5 (KT) + 2.0 (2,4-D)	12

Meanwhile, we collected pre-cotyledons embryos and placed them in IMEC1-IMEC6 mediums (Table 2). With increasing 6-BA and 2,4-D concentrations, the induction percentage of calli was significantly increased, and the highest frequency was 74% in IMEC4 (Table 2). Additionally, this frequency was not improved with the increase in 6-BA and 2,4-D. Therefore, the optimal PGR combinations were determined to be 6-BA 1.2 mg L^−1^ and 2,4-D 2.7 mg L^−1^ for EC initiation.

**Table 2:** Effect of different concentrations of 6-BA, 2,4-D on EC induction from immature zygotic embryo.

Code	Growth regulator(mg/L)		The number of explants	Initiation frequency (%)
	6-BA	2,4-D		
IMEC1	0.6	0.9	100	26.48 ± 0.56^c^
IMEC2	0.6	1.8	100	48.89 ± 2.85^bc^
IMEC3	1.2	1.8	100	56.30 ± 2.16^ab^
IMEC4	1.2	2.7	100	74.00 ± 6.56^a^
IMEC5	1.8	2.7	100	66.83 ± 2.05^ab^
IMEC6	2.4	3.6	100	42.78 ± 7.56^bc^

Bars denote by the same letter within response variables are not significantly different (*P* = 0.05) using Duncan’ s multiple range test.

### Embryos developmental stage is critical for EC induction

Many studies have indicated that the time at which explants are collected plays an important role in EC induction. Moreover, the time window for a positive response has been observed to be short. To obtain the best time window for the SEMs initiation, the seeds were collected from the beginning of fertilization to maturation seeds, which cover the whole developmental period of the zygotic embryo from summer to autumn. Our results indicated that the EC initiation frequency was lower in early July, and gradually increased to 50.1% until late July. It reached to 67.7% in early August. Subsequently, this value gradually decreased to 23.8% and 13.30% in mid and late August, respectively (Table 3). Finally, ECs failed to be induced in early September. Therefore, the best window of time for SEMs initiation was late July to early August.

**Table 3:** Effect of collection dates on EC initiation.

Dates	The number of explants	Initiation frequency (%)
July 5 (about 6 weeks after fertilization)	75	5.56 ± 7.26^ef^
July 15 (about 8 weeks after fertilization)	100	17.00 ± 7.89^ cd^
July 25 (about 9 weeks after fertilization)	95	50.10 ± 10.22^b^
August 5 (about 10 weeks after fertilization)	90	67.70 ± 5.60^a^
August 15 (about 11 weeks after fertilization)	100	23.80 ± 7.44^c^
August 25 (about 12 weeks after fertilization)	95	13.30 ± 7.65^de^
September 5 (about 14 weeks after fertilization)	100	1.37 ± 2.73^f^

Bars denote by the same letter within response variables are not significantly different (*P* = 0.05) using Duncan’ s multiple range test.

Due to the influence of climate and other factors, we attempted to determine which developmental stage of zygotic embryos is the best for SEMs initiation. Based on previous finding^29–31^ and the morphological of megagametophyte, we defined four stages of whole zygotic embryos developmental from early July to mid-September. At the early July, most of embryos were at the pro-embryo stage; they developed into early embryogeny in mid-July. After that, most of them developed into late embryogeny (pre or early cotyledonary embryo) in early August; and maturation stage (mature cotyledon embryos) in later August (Table 4). Therefore, EC initiation frequency was likely positively correlated with the embryo’s developmental stages. Moreover, EC induction frequency was 74% and 12%, when pre-cotyledons and mature cotyledon embryos as explants, respectively. All these results showed that the developmental stage of the initial zygotic embryo is essential for embryo meristem initiation.

**Table 4:** Embryonic developmental stages of different collection dates.

Collection dates	Embryo development phases	Embryo states
July 5	Proembryogeny	Pro embryo
July 15	Early embryogrny	Cleavage poly-embryos
July 25	Early or late embryogrny	Early embryo and pre-cotyledonary embryos
August 5	Late embryogrny	Pre-cotyledonary and early cotyledonary embryos
August 15	Late embryogrny or early maturation	Cotyledonary embryos
August 25	Early maturation	Mature cotyledonary embryos
September 5	Late maturation	Mature embryos and seeds

**Figure 3:**
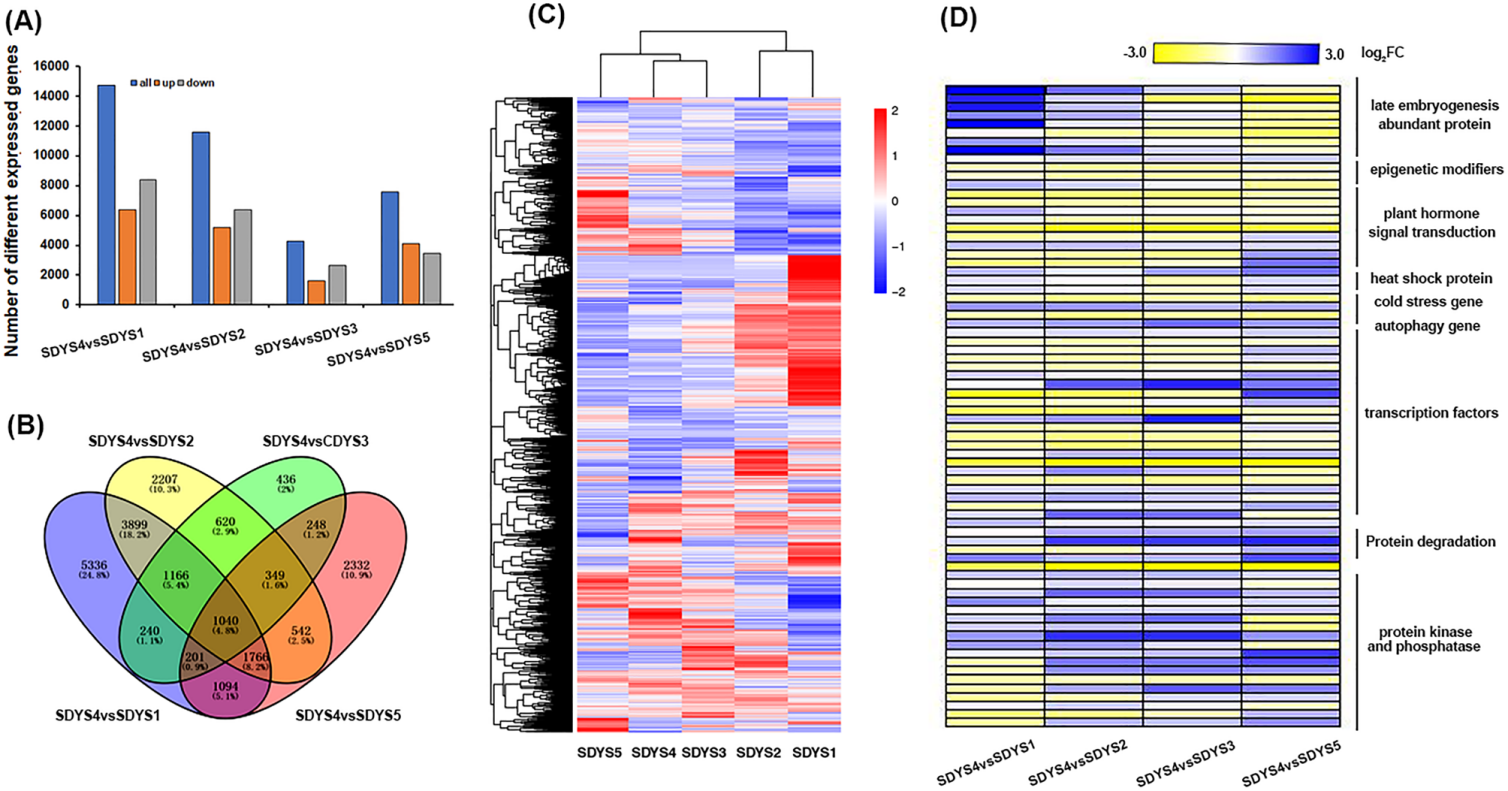
Transcriptome data analysis in different developmental stages of immature zygotic embryos. (**A**) Statistics on the number of differentially expressed genes in early pre-cotyledon stage compared with other development stages. (**B**) Wayne diagram shows the common different expression genes in different groups. (**C**) Heat map of differentially expressed gene clustering figure in different developmental stages of zygotic embryos. (**D**) Heat map of gene expression associated with different signalling processes in zygotic embryos.

To comprehensively analyze the related genes and other molecular markers to characterize into the timing of the response of zygotic embryos to SE, we performed a genome-wide analysis of transcripts using high throughput RNA-seq technology at various development stages. We isolated the embryos at five developmental stages (pro-embryos, cleavage poly-embryos, early embryos, pre-cotyledon, and cotyledon embryos) and sequenced their total RNA. Approximately 190 million raw reads were obtained for each RNA-seq sample (Table S1). The correlation analysis results showed that the repetition of all samples was good (Fig. S1). The generated reads were mapped to the reference genome of *Norway spruce*, and each sample had approximately 80% total mapped reads (Table S1). Moreover, we re-annotated the obtained genes using other spruce databases to obtain supplementary genes information. In total, we found 1,040 differentially expressed genes (DEGs) between pre cotyledon (SDYS4) and the other stages, that is the pro-embryos (SDYS1), cleavage poly-embryos (SDYS2), early embryos (SDYS3) and cotyledon stages (SDYS5), of which 273 DEGs were downregulated and 381 were upregulated in all groups (Fig. 3A–C, Table S1). Among all DEGs, the embryo development related genes were embryo-specific protein ATS3A, and stress response genes were related to late embryogenesis abundant protein LEA (Fig. 3D, Table S3,4). Meanwhile, the transcript levels of epigenetic modification-related genes, histone deacetylases and numerous hormone response/transduction and biosynthesis-associated genes were upregulated in SDYS4. The transcript levels of ten plant hormone related genes, such as auxin-induced protein 6B, auxin efflux carrier and IAA10; ABA signaling pathway genes PP2C, SnRK, and NCED9; and gibberellin pathway genes GA2ox12 and gibberellin-regulated protein 11, were upregulated in SDYS4. In contrast, the transcript levels of autophagy-related protein 18 and the F-box protein were down-regulated in this stage. In addition, transcription factor, such as NAC56, WRKY1, AP2, bHLH1, and the MYB family (MYB4, 5, 29, 32, 38, 86) transcription factors were differently expressed in SDYS4. Therefore, these genes may be involved in SEMs initiation in *P. mongolica.*

### Plant growth regulators are critical in somatic embryogenesis

**Figure 4:**
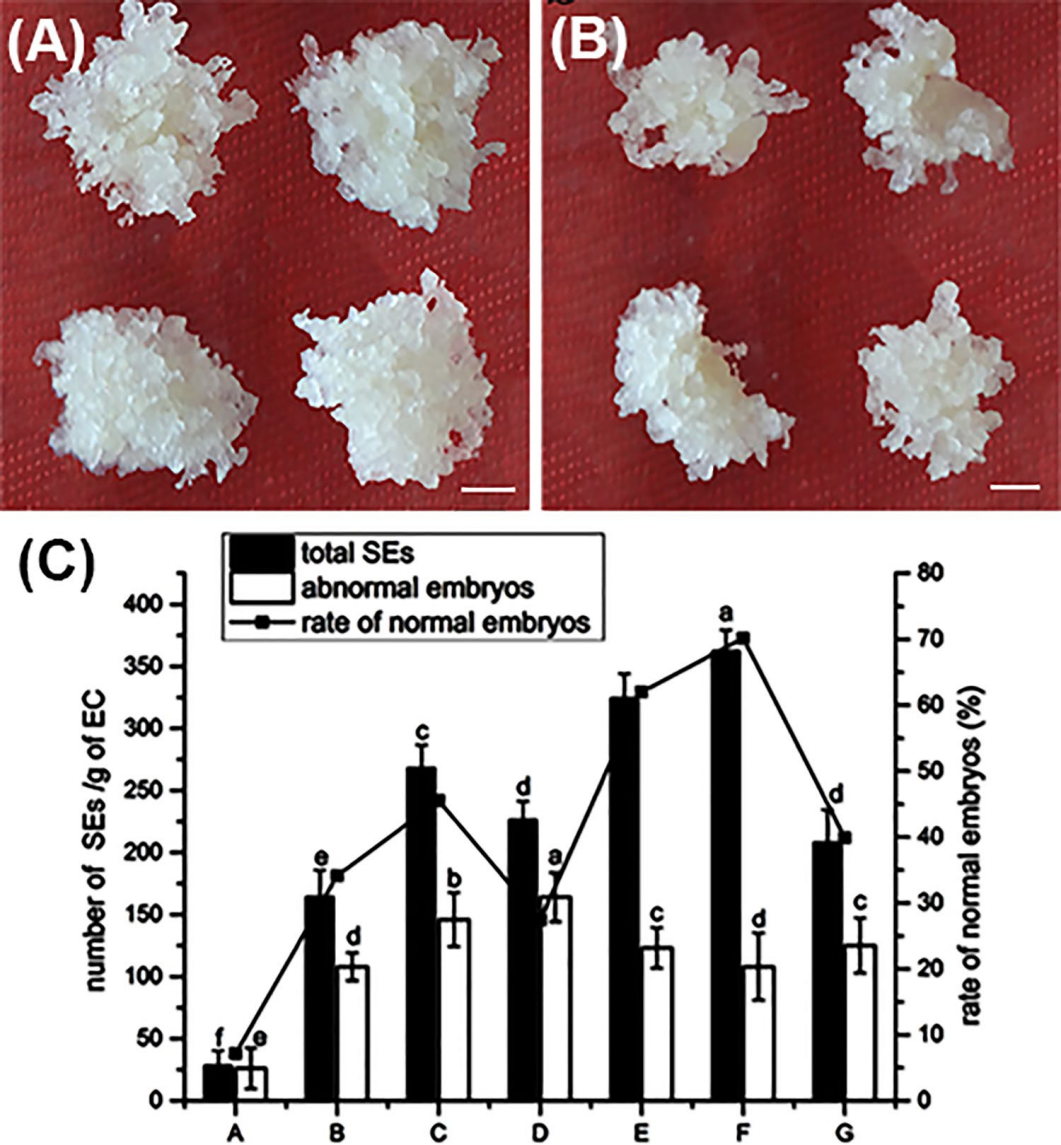
Effect of plant growth regulators on EC proliferation and SEMs maturation. (**A**, **B**) Effect of 2,4-D on EC proliferation. (**A**) EC proliferation on 1/2 MS medium with 1.6 mg L^−1^ 2,4-D, and the fresh weight grew quickly. (**B**) EC proliferation on 1/2 MS medium with 3.2 mg L^−1^ 2,4-D, and the fresh weight grew slowly. Bars 1 mm. (**C**) Effect of different concentrations of ABA and PEG4000 on SEMs maturation. The X axis represents different concentrations of ABA and PEG4000, A, B, C, and D only add ABA at 7.5 mg L^−1^, 15 mg L^−1^, 22.5 mg L^−1^ and 30 mg L^−1^ respectively, to 1/2 LM media. E, F and G add 22.5 g L^−1^, 45 g L^−1^, 67.5 g L^−1^ PEG4000 on 1/2 LM with 22.5 mg L^−1^ ABA. Bars denoted by the same letter within response variables are not significantly different (*P* = 0.05) using Duncan’s multiple range test. The experiment was repeated three times.

When the ECs were transferred into the proliferation medium (PM), some white or pale yellow, shiny, and soft small nodular structures appeared on the callus surface (Fig. 4A,B). The statistical results demonstrated that the fresh weight of obtained ECs was slightly increased approximately only 1.00 g after transfer cultivation for 4 weeks proliferation (Table 5), suggesting that 6-BA could not significantly improve ECs proliferation effectiveness. However, 2,4-D had a positively function in ECs proliferation (Fig. 4A,B). The fresh weight of ECs was increased by 3.87 g in PM with 1.6 mg L^−1^ 2,4-D after 4 weeks; and its reproduction percentages were 5.91 (Table 5). However, after increasing 2,4-D to 3.2 mg L^−1^, the growth weight was only 2.53 g, and the reproduction percentage was 3.61 (Table 5). Therefore, 2,4-D but not 6-BA is critical for the subsequent ECs proliferation in SE of *P. mongolica*.

**Table 5:** Effect of different concentrations of 6-BA and 2,4-D on EC proliferation.

6-BA (mg L^−1^)	Early inoculation (g)	Four weeks after inoculation (g)	Multiplication rate	2,4-D (mg L^−1^)	Early inoculation (g)	Four weeks after inoculation (g)	Multiplication rate
0.8	0.53 ± 0.06^a^	1.54 ± 0.35^a^	1.91 ± 0.56^a^	0.8	0.71 ± 0.12^a^	4.58 ± 0.53^a^	5.45 ± 1.10^a^
1.6	0.62 ± 0.07^a^	1.60 ± 0.28^a^	1.58 ± 0.33^ab^	1.6	0.59 ± 0.15^a^	4.08 ± 0.86a^b^	5.91 ± 1.86^a^
2.4	0.62 ± 0.07^a^	1.49 ± 0.38^a^	1.40 ± 0.46^b^	2.4	0.69 ± 0.11^a^	3.53 ± 0.75^bc^	4.12 ± 0.97^b^
3.2	0.57 ± 0.09^a^	1.37 ± 0.27^a^	1.40 ± 0.42^ab^	3.2	0.70 ± 0.11^a^	3.23 ± 0.68^c^	3.61 ± 0.90^b^
Mean	–	–	–	–	–	–	***

Bars denote by the same letter within response variables are not significantly different (*P* < 0.05) using Duncan’ s multiple range test. The results are significantly different (*P* < 0.01) denoted with ***by Paired t test.

During the maturation process, the statistical results demonstrated that the maturation of somatic embryo was strongly suppressed, which led to abnormal somatic embryos at relatively lower concentrations of 10 mg L^−1^ ABA. The number of SEMs gradually increased to less than 22.5 mg L^−1^, but decreased to more than 30 mg L^−1^ with the reduced of ABA concentrations (Fig. 4C). The maximum number of 268 embryos was obtained in the maturation medium with 22.5 mg L^−1^ ABA (Fig. 4C). These results showed that ABA promotes the growth and development of SEMs at relatively lower concentrations of 15–30 mg L^−1^. To further improve the yield of early SEMs, we simultaneously used 22.5 mg L^−1^ ABA and different concentrations of PEG4000. The yield of early SEMs was dramatically increased; but when PEG4000 was more than 45 g L^−1^, its percentage was increased (Fig. 4C). We obtained 362 differentiated somatic embryos in total. Among them, abnormal embryos were 108/g, and its percentage of normal SEMs was 70.17% (Fig. 4C). Therefore, utilizing optimal concentrations of ABA (22.5 mg L^−1^) and PEG4000 (45 g L^−1^) could promote the maturation of *P. mongolica* somatic embryos.

### Genetic controls ECs induction and somatic embryogenesis

**Figure 5:**
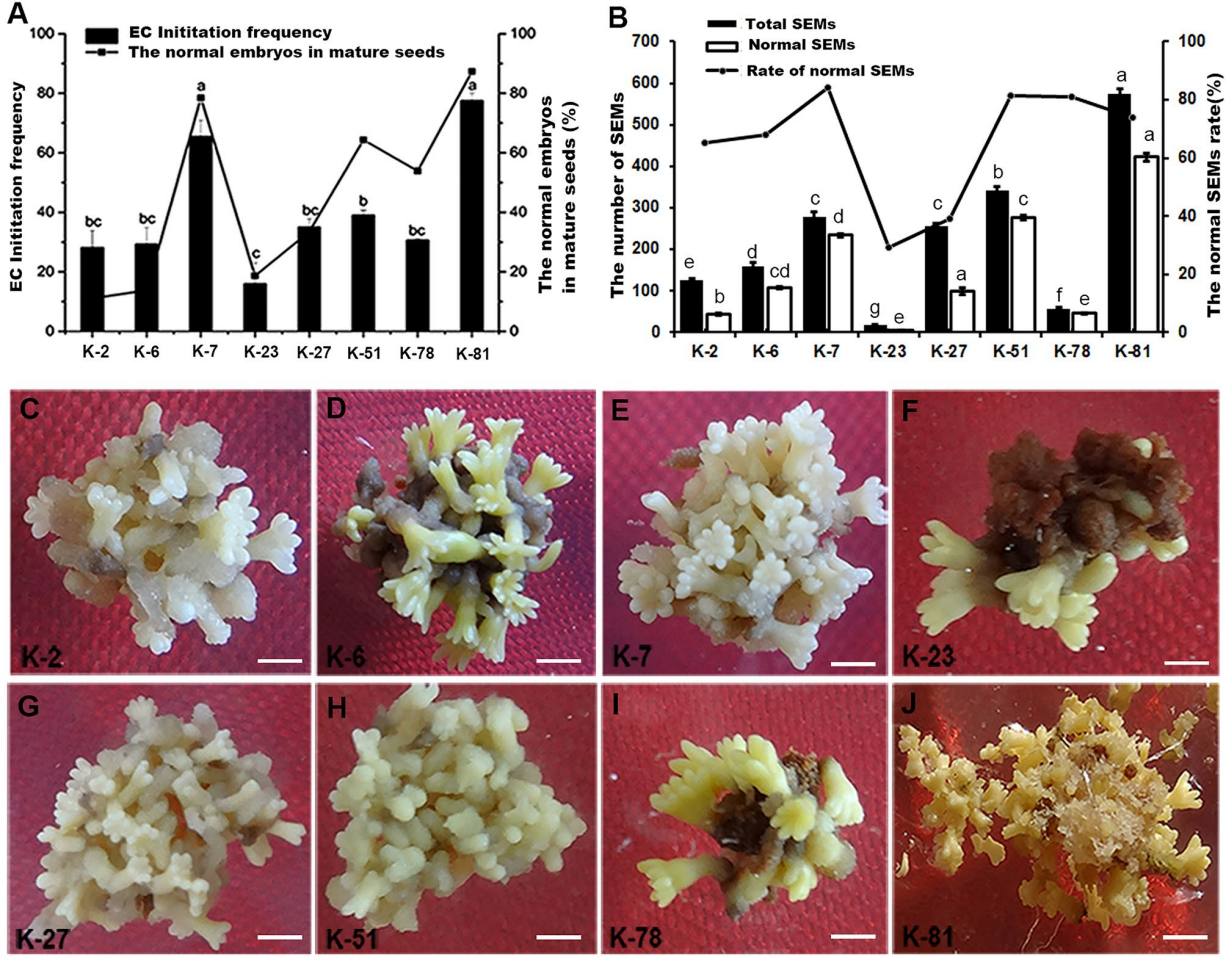
Effect of the maternal genotypes of donor plants on EC induction and SEMs maturation. (**A**) Effect of different lines and the seed filling rate on EC initiation. Bars denoted by the same letter within response variables are not significantly different (*P* = 0.05) using Duncan’ s multiple range test. (**B**) The total number of generated SEMs and normal SEMs frequency in the different donor lines. (**C**–**J**) The SEMs maturation from different donor lines. The mature cotyledon embryos are milk white that obtained from K-2, K-7, K-27 and K-51. K-6, K-23 and K-78 generated mature cotyledon embryos were pale-yellow and same browned abnormal embryos. The embryos produced by K81 are pale-yellow with mature cotyledon embryos. Bars: 1 mm. All experiments were repeated more than three times.

The frequency of EC induction is dramatically affected by the maternal tree genotype. Due to the poor growth environment of *P. mongolica*, abnormal embryos are very obvious in mature seeds. We speculated that the rate of normal embryos in each cone were attributed to their maternal tree genotypes. As shown in Fig. 5A, the highest EC induction frequency (77.50%) was observed in K-81, and its percentage of seeds with normal embryos was 87.34%, while the worst percentage was only 15.91% in K-23. Therefore, EC induction may be positively correlated with the ratio of normal embryos in each cone. However, the frequency in K-2 and K-78 was the same (30%), but their abnormal embryos rate was different 13.77% and 53.91%, respectively (Fig. 5A). Therefore, the genetic factors significantly control the EC induction in SEMs initiation. But the ratio of normal embryos cannot represent the superiority of genotype in their mother trees.

In addition, the maternal tree genotypes were also significantly associated with the frequency of SEMs mutation. Among the eight donor plants, K-81 was the best responsive genotype for SEMs generation with 573.4 SEMs per gram, whereas K23 was the worst with 16 per gram (Fig. 5B). Most of the SEMs with early and late cotyledonary stages generated from K-2, K-7, K-27 and K-51 were white, translucent bulges emerging from the surface of ECs, while the SEMs from K81, K-2, K-7, K-27 and K-51 were pale-yellow and exhibited smooth texture, as well as more developed SEMs (Fig. 5C–J). The embryos produced from K-81 were widely scattered around the tissues and located at the junction of tissues and culture medium (Fig. 5J). In other donor plants, although embryos that emerged from the surfaces of ECs with tip-shaped cotyledon bulges, they were recalcitrant for further development and gradually began to turn dark brown and necrotic (Fig. 5D,F,I). Moreover, many abnormal embryos, such as those with abnormally swollen hypocotyls, multiple cotyledons or monocots, and two fused embryos, were often observed during SEMs maturation. Among the eight mother trees, the highest percentage normal of SEMs was observed in K-7 (83.94%), and obtained total SEMs was 340 per gram (Fig. 5B). Although the percentage of normal SEMs was 73.7%, the most of SEMs were obtained in K-81. Therefore, the best responsive genotype tree was K-81.

## Discussion

SE is a multistep regeneration process that is involved in ECs initiation, proliferation, SEMs maturation and plantlet regeneration^32–35^. ECs can be generated from a variety of tissues, such as zygotic embryos and vegetative shoot apices in coniferous species. Although zygotic embryos are the best explant, the initiation of SEMs is limited to a developmental window of several weeks, during which the embryos are highly competent for SE in most pine species^35,36^. Previous study show that its induction frequency is 13% in *P.pungens*^32^, 8% in *P. mariana*^20^, and is very low in *P. mongolica*^36^ using mature embryos as explants. The greatest success in inducing SE in coniferous species has occurred with immature zygotic embryos. The frequency with which explants developed into ECs was significantly affected by the developmental stage of the immature embryos. In this study, the ECs yield was also lower when mature embryos are used than when immature embryos were used. Its induction rate was 12% and 74%, respectively. We found that the best response time for EC induction was from late July to early August, about 9–10 weeks after fertilization. Moreover, the later embryogeny ranging from pre-cotyledon to early cotyledon embryo stage were the most responsive to induction of the ECs for initiation of SEMs, which is similar to the reports that pre-cotyledon embryos in later embryogeny were benefit for SE, while cotyledonary stage embryos were less successful in pine species^14,37,38^.

Previous reports indicate that hundreds of genes have been directly linked to zygotes and SE; some of them, such as SOMATIC EMBRYOGENESIS LIKE KINASE (SERK), LEAFY COTYLEDON (LEC), BABYBOOM (BBM), and AGAMOUSLIKE 15 (AGL15) are very important part of the molecular network^39^. In here, our transcriptomic analysis also reveals that several stress-related transcription factors and hormone signalling, including auxin, ABA and ethylene pathways might contribute to the differentiation of EC. This is similar to the results that the auxin and stress hormone responses are involved in the callus dedifferentiation and trans differentiation processes in cotton somatic embryogenesis^40,41^. Additionally, epigenetic modification-related genes were determined to be enriched in different groups. The reduction of DNA methylation level perhaps triggers cell dedifferentiation through the expression of related genes in SE processes^42^.

PGRs play an important role in the processes of EC initiation, proliferation, and SEMs maturation^43^. Auxin and cytokinin are the key regulators of plant cell division and differentiation, which promote somatic cells to acquire embryogenic competence^44^. A previous study indicates that PGRs are also associated with initiation and proliferation of ECs in coniferous species^45^. In our study, we developed two efficient SE systems with zygotic embryos explants. Our results indicates that distinct media differences are important for successfully capturing ECs. The high concentration of PGRs could induce EC generation from immature and mature zygotic embryos. Combined use of 2,4-D, 6-BA significantly enhances induction of *P. mongolica* ECs, but higher concentrations of auxin/cytokinins could not significantly improve the frequency of ECs in medium EMC3-6 supplemented with NAA and 6-BA. In the present study, we found that KT is essential for mature embryos EC induction. In our initial culture, although the induction rate increased to 78%, the callus readily developed to be a brown color. Subsequently, we transferred the generated ECs to continue culture in EMC9 with low concentrations of 2,4-D, 6-BA and KT. The highest frequency of ECs (12%) induction was obtained from MEC9. For this reason, we surmise that a high concentration of 2,4-D has a toxic effect producing of embryogenic tissue at the late stage of callus development, but a low concentration of KT could antagonize 2,4-D to inhibit callus browning. This phenomenon may be consistent with the result that auxin and KT antagonistically regulate DNA methylation modification to affect callus development in *Daucus carota*^46^. Moreover, our RNA-seq results indicate that the transcript levels of epigenetic modification-related genes, histone deacetylases are upregulated in the pre-cotyledon stage.

Early somatic embryos may rapidly accumulate in medium supplemented with appropriate concentrations of ABA and PEG4000^47^.Stress is recognized as a signal that directs plant cells to undergo reprogramming (dedifferentiation) as a means for adaptation and in preparation for a stimulus-based acquisition of a new cell fate^48^. ABA is the key factor that increases the yield of somatic embryos in angiosperms and gymnosperms during SEMs maturation^48,49^. Previous studies confirm that somatic embryo development must be stimulated by exogenous ABA, which concomitantly reduces cell proliferation during SE processes in most coniferous species^47,50,51^. PEG supplementation is an important osmotic adjustment for maintaining the differentiation of cells in a relatively moderate infiltration environment, thereby dramatically improving the quality of SEMs^52–54^. In the present study, we found that the highest yield of early somatic embryos was generated in medium with ABA (22.5 mg L^−1^) and PEG4000 (45 g L^−1^).

The success of regenerating plants via SE is largely dependent on the genotype of the plant species^55^. A previous study indicated that different plant complements respond differently^56,57^. Marked differences in the responses of several pine species were reported that genotypes can behave differently under different conditions. The former being non-or partially responsive to induction of SEMs when similar experimental approaches were applied^58^. Our data show that the produced EC is also under strong genetic control in *P. mongolica*. The seed families from eight genotypes of mother trees responds significantly differently. The frequency of EC induction was significantly higher in the mother trees K-81 than other trees. This is consistent with previous studies in *Picea glauca* and *Picea mariana*^34,35^*.*

In this study, we successfully established an efficient EC development system from immature and mature zygotic embryos in *P. mongolica*. Multipe affecting the efficiency of SE were comprehensively analyzed. High quality and abundant mature somatic embryos were obtained from embryogenic tissues induced in maturation medium containing optimal concentrations of growth regulators. However, the molecular mechanism of PGRs and the dominant maternal genotype regulating EC initiation, EC proliferation, and SEMs maturation should be further deeply researched. These topics will be our further research directions for the regeneration of *P. mongolica*. In summary, our SE development system will establish a foundation for in-depth analysis of molecular mechanisms, which could be used as a tool for improving Chinese *P. mongolica* at the molecular level by means of genetic engineering. Meanwhile, the method has significant application value for the protection of *P. mongolica* spruce germplasm resources and the expansion of Chinese natural spruce resources.

## Methods

### Plant material

To obtain an efficient plant regeneration system for *P. mongolica*, zygotic embryos from twenty-year-old trees were used as explants. Healthy seeds were collected from the Baiyinaobao nature reserve in Inner Mongolia, China. In spring 2015, immature seeds were collected every ten days from July 5 to September 5 between 6 and 15 weeks after fertilization. Approximately 30 cones from 8 maternal trees (K-2, K-6, K-7, K-23, K-27, K-51, K-78, K-81) with high seed production were obtained. Mature seeds were collected in October of the same year.

Immature and mature seeds were disinfested in 75% (w/v) ethanol for 30–40 s followed by being washed in tap water three times and being shaken in 2% (v/v) NaClO for 6–12 min. After rinsing three times in sterile water, their zygotic embryos were stripped out aseptically from megagametophytes with a scalpel and tweezers. For EC induction, mature and immature embryos as explants. The mature cotyledonary embryo from mature seeds were obtained in October, 2015. The immature zygotic embryos obtained from immature seeds containing different development stage embryos at 6 and 14 weeks after fertilization at the same year. The normal embryos rate was counted in each cone (normal embryos seeds/ all seeds in each cone). Thirty cones per maternal trees were analyzed. The embryos were placed horizontally onto a disposable sterile Petri dish with embryogenic tissue induction medium as the initial explants for the subsequent somatic embryogenesis assay.

### Embryogenic cultures induction

When mature embryos were used as explants, mature cotyledon embryos were cultured on the callus induction medium, which contains basal salts reduced to half the concentration of the standard medium^37^. All media were supplemented with 20 g L^−1^ sucrose, 2.5 g L^−1^ gellam gum (Phytagel), 700 mg L^−1^ casein acid hydrolysate and 400 mg L^−1^ glutamine with different concentrations of PGRs (Table 1). The pH was adjusted to 5.8 prior to autoclaving the medium for 15 min at 121 °C. All jars were incubated in the dark for 14 days at 25 ± 1 °C and then transferred to the growth chamber under the aforementioned conditions. Each diametric petri dishes containing sterile induction medium was inoculated with 5 explants, and each treatment had 10 replicates. The number of produced calli and EC initiation percentage [(callus or EC-producing explants/total explants) × 100%] were recorded every 10 days. All EC induction assays were performed in triplicate.

The immature pre-cotyledon embryos were placed on 1/2 Litvay Medium (LM) supplemented with 20 g L^−1^ sucrose, 2.5 g L^−1^ gellam gum (Phytagel), 700 mg L^−1^ casein acid hydrolysate, and 400 mg L^−1^ glutamine as previous study^37^. Here, different concentrations of the plant growth regulators 6-BA (0.6 mg L^−1^, 1.2 mg L^−1^, 1.8 mg L^−1^, 2.4 mg L^−1^) and 2,4-D (0.9 mg L^−1^, 1.8 mg L^−1^, 2.7 mg L^−1^, 3.6 mg L^−1^) (Table 2) were used to determine the efficiency of embryogenic tissue induction. The explants were incubated in medium for 25 days in the dark at 25 ± 1 °C. Each diametric petri dishes containing sterile induction medium was inoculated with 5 explants, and each treatment had 10 replicates. The number of explants producing EC was recorded. Three replicates were used per each induction treatment.

To test the effect of genotype on the induction of ECs, we used 1/2 LM with the same quantity of sucrose, phytagel, casein acid hydrolysate, and glutamine. Based on the above basic medium, we supplemented 1.2 mg/l 6-BA and 2.7 mg/l 2,4-D, which are the optimal concentrations obtained the highest EC initiation frequency. We randomly selected eight donor plants and to obtain zygotic embryos at the pre-cotyledonary stage to detect the frequency of EC induction in the maternal tree genotype. Each diametric petri dishes containing sterile induction medium was inoculated with 5 explants, and each treatment had 10 replicates. Each induction treatment was performed with three replicates.

### Embryogenic cultures proliferation

In EC proliferation, 1/2 LM mediums containing 20 g L^−1^ sucrose, 2.5 g L^−1^ gellam gum (phytagel), 700 mg L^−1^ casein acid hydrolysate, and L-glutamine 400 mg L^−1^ with different concentrations of the plant growth regulators 6-BA (0.8 mg L^−1^, 1.6 mg L^−1^, 2.4 mg L^−1^, 3.2 mg L^−1^) and 2,4-D (0.8 mg L^−1^, 1.6 mg L^−1^, 2.4 mg L^−1^, 3.2 mg L^−1^) were used in Table 5. The pH was adjusted to 5.8 prior to autoclaving. The embryogenic cultures were incubated in PM medium for 4 weeks in the dark at 25 °C. Each bottle of culture medium was inoculated with 5 explants, and each treatment had 10 replicates. We separately recorded the weight of initial inoculation and that after 4 weeks proliferation. The effectiveness of EC proliferation [(weight after proliferation 4 weeks-weight of initial inoculation)/weight of initial inoculation] was recorded. All the assays were performed with three replicates.

### Maturation of somatic embryos

To initiate the maturation phase, the cytokinins and auxin in the 1/2 LM containing 20 g L^−1^ sucrose, 2.5 g L^−1^ gellam gum (Phytagel), 700 mg L^−1^ casein acid hydrolysate and L-glutamine 400 mg L^−1^ medium was substituted with ABA (Sigma-Aldrich) and polyethylene glycol 4000 as described in a previous study^16^. The proliferated EC were cultured in clumps with different concentrations of ABA (7.5 mg L^−1^, 15 mg L^−1^, 22.5 mg L^−1^, 30 mg L^−1^) and PEG4000 (22.5 g L^−1^, 45 g L^−1^, 67.5 g L^−1^) for 4–5 weeks. The pH was stabilized at 5.8, and the plates were sealed with Parafilm in darkness at 25 °C. The number of SEMs and percentage of normal SEMs [(number of total SEMs—abnormal embryos)/number of total SEMs × 100%] were recorded for each treatment. All the assays were performed with three replicates.

### Somatic embryo desiccation

After 4 weeks of maturation, fully developed embryos were selected and desiccated. The embryos were carefully transferred onto dry paper, in small Petri dishes. Several paper layers wetted with sterile water (100% humidity) were place under dry filter paper. The dishes were covered with lids, sealed with parafilm, and kept under darkness, at 25 °C for 1 week. Then they continue to dry in the light condition with 16 h photoperiod (16 h light (PPFD approximately 15–25 μmol m^−2^ s^−1^, daylight fluorescent tubes and 8 h darkness) for 1 week.

### Somatic embryos germination

Desiccated embryos were collected and transferred into germination medium (1/2 LM containing 20 g L^−1^ sucrose, 2 g L^−1^ active charcoal), pH 5.8. Dishes with embryos were placed in a cultivation room at 25 °C under a photon flux density of 45–65 µmol m^−2^ s^−1^ provided by 100 V, 40 W white fluorescent lamps for 2 weeks. The photoperiod was 16 h (16 h light and 8 h darkness). After germination, the root and epicotyl emergence with 1-week dark culture; cotyledons are produced after 2 weeks on the light. Subsequently, they continue to grow for 2 weeks in incubator at 25 °C. After that, they were transplanted into the containers filled with substrate (volume of peat: perlite: roseite = 3:1:1) for further cultivation.

### Paraffin section assay

To detect SEMs development, ECs and SEMs tissues at different developmental stages were obtained and immediately fixed with 10% FAA solution. FAA solution was washed off and the samples were stained with Ehrlich’s haematoxylin staining solution (hematoxylin 2 g, glacial acetic acid 10 ml, glycerol 100 ml, 95% ethanol 100 ml, distilled water 100 ml, aluminum potassium sulfate 5 g). The samples were embedded in paraffin after dehydration and transparency. Next, the tissue was observed, and pictures were taken after sectioning.

### Developmental stages of the zygotic embryos

For testing the effects of different embryonic development stages on the induction of EC, we obtained the embryo after fertilization from July to September. The developmental stages of the zygotic embryos were evaluated at each collection date, which cover the whole developmental period of seed development. 50 randomly chosen seeds were dissected with a scalpel and tweezers, and the embryos were examined under a stereomicroscope. We divided them with four stages, proembryony, early embryogeny, late embryogeny, and maturation according as a previously reported embryogeny staging system in conifer^23^.

### Statistical analysis

All experiments were repeated at least three times. The graphical representations of data were made using Origin Pro version 9.0 software to obtain a graph of data (Origin Lab, Northampton, MA, USA). The SPSS Windows version 21 (SPSS Inc., Chicago, IL, USA) was used for variance analysis. The data were analyzed with one-way ANOVA to calculate statistical significance, and Duncan’s new multiple range test was employed for comparison among treatment means.

### RNA isolation, sequencing, and data analysis

According to microscope observations, we divided the zygotic embryos samples and named SDYS1, SDYS2, SDYS3, SDYS4, SDYS5 based on the embryonic developmental stages. Total RNA was isolated separately from five zygotic embryo development stages using the MiniBEST Plant RNA Extraction Kit (Takara, Dalian, Liaoning, China) according to the manufacturer’s protocol. We selected three biological replicates for sequencing at each developmental stage. The sequencing data have been deposited in the NCBI Gene Expression Omnibus (GEO) database. The RNA-seq reads that passed quality control were aligned to the reference genome of Norway spruce. Differential expression analyses of the four groups were performed using the DESeq2 R package (1.16.1). Thresholds of |log2 (fold change) |≥ 1 and an adjusted padj < 0.05 were considered to indicate a significant difference in expression. Gene Ontology (GO) enrichment analysis of differentially expressed genes (DEGs) was performed using the GOseq R package. KOBAS software was used to test the enrichment of DEGs in KEGG pathways.

## Supplementary Information

Supplementary Information 1.

Supplementary Information 2.

Supplementary Information 3.

Supplementary Information 4.

Supplementary Information 5.
