# Supplementary figures and images for: Multiple analyses of various factors affecting the plantlet regeneration of Picea mongolica (H. Q. Wu) W.D. Xu from somatic embryos
Source: Sci Rep. 2021 Mar 23;11:6694. doi: 10.1038/s41598-021-83948-w (PMC7987962; doi:10.1038/s41598-021-83948-w)

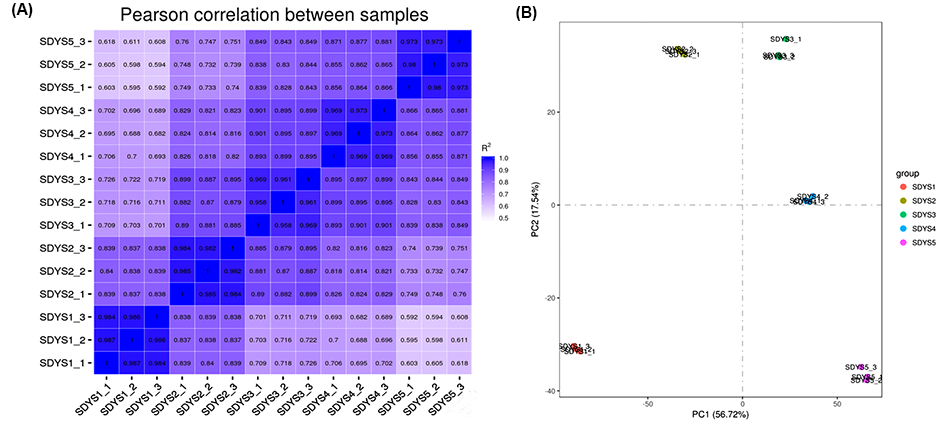

Supplement: Supplementary file 5 — Supplementary Information 5. [file 41598_2021_83948_MOESM5_ESM.tif]
